# Impact of community-based interventions on HIV: the next steps

**DOI:** 10.1186/2049-9957-3-34

**Published:** 2014-09-17

**Authors:** Kieran Walsh

**Affiliations:** 1 BMJ Learning, BMA House, Tavistock Square, London WC1H 9JR, UK

**Keywords:** Community, Education, HIV

## Abstract

Community based interventions increase knowledge scores and also have an impact of sexual behaviours with regard to HIV. However the problem remains as to how best to scale up these interventions and how best to overcome real or perceived barriers to their uptake. Community based interventions have multiple components and some will be more difficult to widen out than others. Those that involve face to face or one to one sessions will be most expensive and so most difficult to scale up. If some interventions can be implemented by means of custom computerized risk reduction programmes, then roll out on a large scale should be less problematic.

## Multilingual abstracts

Please see Additional file 1 for translations of the abstract into the six official working languages of the United Nations.

## Dear Editor

Salam et al. should be congratulated for presenting the results of an insightful systematic review describing the impact of community based interventions on HIV knowledge, attitudes, and transmission [1]. And the results of the review are clear - community based interventions increase knowledge scores and also have an impact of sexual behaviours. However the problem remains as to how best to scale up these interventions and how best to overcome real or perceived barriers to their uptake. With the massive numbers of patients who are infected or who are at risk, it is likely that scale up is what will make a real difference in the next decade.

As with all attempts to scale up, success will be decided by the actual intervention or the component or components of an intervention that is being scaled up. Community based interventions have multiple components and some will be more difficult to widen out than others. Those that involve face to face or one to one sessions will be most expensive and so most difficult to scale up. If some interventions can be implemented by means of custom computerized risk reduction programmes, then roll out on a large scale should be less problematic. The continuing widening availability of the internet in all parts of the world surely offers significant opportunity in this regard. Mobile technology now means that simple programmes can be made available via smartphones. Advantageously the younger population who are most at risk are likely to be most adept at using such new technology.

Community based interventions could also help to overcome one of the most substantial barriers to the uptake of community based interventions â€“ the real or perceived need to conform with traditional cultural practices and beliefs, and continuing intrinsic reluctance to learn and talk about sex in public. Internet based interventions can allow individuals to learn about sex and sexual health in private. Perhaps even more importantly they can allow individuals to submit their questions and receive answers anonymously. This latter capability could allows at risk individuals to take a more active and interactive role in learning about HIV infection â€“ and active learning is much more likely to be effective learning.

Community based interventions are undoubtedly effective â€“ however new technologies might enable them to reach much larger numbers of affected or at risk people.

Yours Sincerely,

Dr Kieran Walsh

## Competing interests

The authors declare that they have no competing interests.

## Supplementary Material

Additional file 1 Multilingual abstracts in the six official working languages of the United Nations. Click here for file
