# Supplementary material for: Impact of community-based interventions on HIV: the next steps
Source: Infect Dis Poverty. 2014 Sep 17;3:34. doi: 10.1186/2049-9957-3-34 (PMC4172394; doi:10.1186/2049-9957-3-34)
Supplement: Additional file 1 — Multilingual abstracts in the six official working languages of the United Nations. [file 2049-9957-3-34-S1.pdf]

Translation of the abstract into the six official working languages of the United Nations

تأثير التدخلات المجتمعية على فيروس نقص المناعة البشرية HIV: الخطوات القادمة

كيران ولش

ملخص

تزيد التدخلات المجتمعية من معدلات المعرفة وتؤثر كذلك في السلوكيات الجنسية فيما يخص فيروس نقص المناعة البشرية HIV. ولكن تبقى المشكلة لأفضل السبل لتوسيع نطاق هذه التدخلات وأفضل السبل للتغلب على العوائق الواقعية أو المتصورة لاستيعابها. تحتوي التدخلات المجتمعية على عناصر متعددة وبعضها سيكون أكثر صعوبة في توسيع نطاقه من غيره. فتلك التدخلات التي تستلزم جلسات فردية أو وجهاً لوجه ستكون تكلفتها أعلى ومن الصعب جداً توسيع نطاقها. إذا كان يمكن تنفيذ بعض التدخلات عن طريق برامج الحاسوب المصممة للحد من المخاطر، حينئذ سيكون الطرح على نطاق واسع أقل إثارة للجدل.

Translated from English version into Arabic by Saher Salama, through

促进社区为基础的 HIV 干预效果的后续措施

Kieran Walsh

摘要

以社区为基础的干预措施可以提高高危人群对预防 HIV 相关知识的了解，减少不安全性行为。但是，如何进一步促进干预效果的实施，使得干预效果能够真正被接受并持续发挥作用仍然是一个问题。社区为基础的干预措施往往是有针对性的综合措施，有些措施不适合大范围的推广。比如，面对面的或者一对一的现场干预措施，不但成本高昂而且无法长期持续和大范围推广。如果一些干预措施可以通过计算机或手机等现代化的交流工具实现，那么大范围的推广实施将不再是问题。

Translated from English version into Chinese by Tian Li-guang, through

Impact des interventions communautaires sur le VIH: les prochaines étapes

Kieran Walsh

Sommaire

Les interventions communautaires augmentent le niveau des connaissances et ont également un impact sur les comportements sexuels en ce qui concerne le VIH. Reste cependant le problème d'amplifier au maximum ces

interventions et de surmonter au mieux les obstacles perçus ou réels pour leur diffusion. Les interventions communautaires ont de multiples composantes, dont certaines seront plus difficiles à élargir que d’autres. Celles qui comportent des séances de “face à face” ou de “one to one” seront très chères, donc plus difficiles à amplifier. Si certaines interventions peuvent être mises en œuvre au moyen de programmes informatiques de réduction des risques, alors le déploiement à plus grande échelle devrait être moins problématique.

Translated from English version into French by Ode Laforge, through

Влияние территориальных мер на ВИЧ: следующие шаги

Киеран Уолш

Отрывок

Территориальные меры повышают уровень осведомленности и влияют на половое поведение в отношении ВИЧ. Однако остается актуальным вопрос о том, как наилучшим образом увеличить количество этих мер и преодолеть имеющиеся или предполагаемые барьеры на пути их внедрения. Территориальные меры включают в себя множество элементов, некоторые из которых сложнее расширить, чем другие. Те меры, которые предполагают проведение индивидуальных или тет-а-тет сеансов, будут самыми дорогими, и их будет сложнее всего нарастить. Если некоторые меры можно будет внедрить посредством специальных компьютерных программ для снижения степени риска, то их масштабная реализация станет менее затруднительной.

Translated from English version into Russian by tatiana_com, through

Intervenciones sobre el VIH basadas en la comunidad: siguientes pasos

Kieran Walsh

Resumen

Las intervenciones basadas en la comunidad incrementan los índices de conocimiento, además de tener un impacto sobre el comportamiento sexual en relación con el VIH. No obstante, el problema sigue radicando en los intentos de perfeccionar estas intervenciones y en la búsqueda de la mejor manera de superar barreras, reales o aparentes, a su acogida. Las intervenciones basadas en la comunidad tienen múltiples componentes, de los cuales algunos será más difícil de ampliar que otros. Aquellas que implican sesiones cara a cara o individualizadas serán más caras y, por

tanto, más difíciles de mejorar. Si algunas de estas intervenciones pudieran llevarse a cabo a través de programas informáticos personalizados de reducción de riesgos, el lanzamiento a gran escala debería ser menos problemático.

Translated from English version into Spanish by Nothomb, through

TRANSLATORS
WITHOUT BORDERS
